# Supplementary material for: LC3B is an RNA-binding protein to trigger rapid mRNA degradation during autophagy
Source: Nat Commun. 2022 Mar 17;13:1436. doi: 10.1038/s41467-022-29139-1 (PMC8931120; doi:10.1038/s41467-022-29139-1)
Supplement: Supplementary file 1 — Supplementary Information [file 41467_2022_29139_MOESM1_ESM.pdf]

Supplementary Information

LC3B is an RNA-binding protein to trigger rapid mRNA degradation during autophagy

Hyun Jung Hwang, Hongseok Ha, Ban Seok Lee, Bong Heon Kim, Hyun Kyu Song, and Yoon Ki Kim*

*To whom correspondence should be addressed. E-mail: yk-kim@korea.ac.kr; Tel.: +82 2-3290-3410

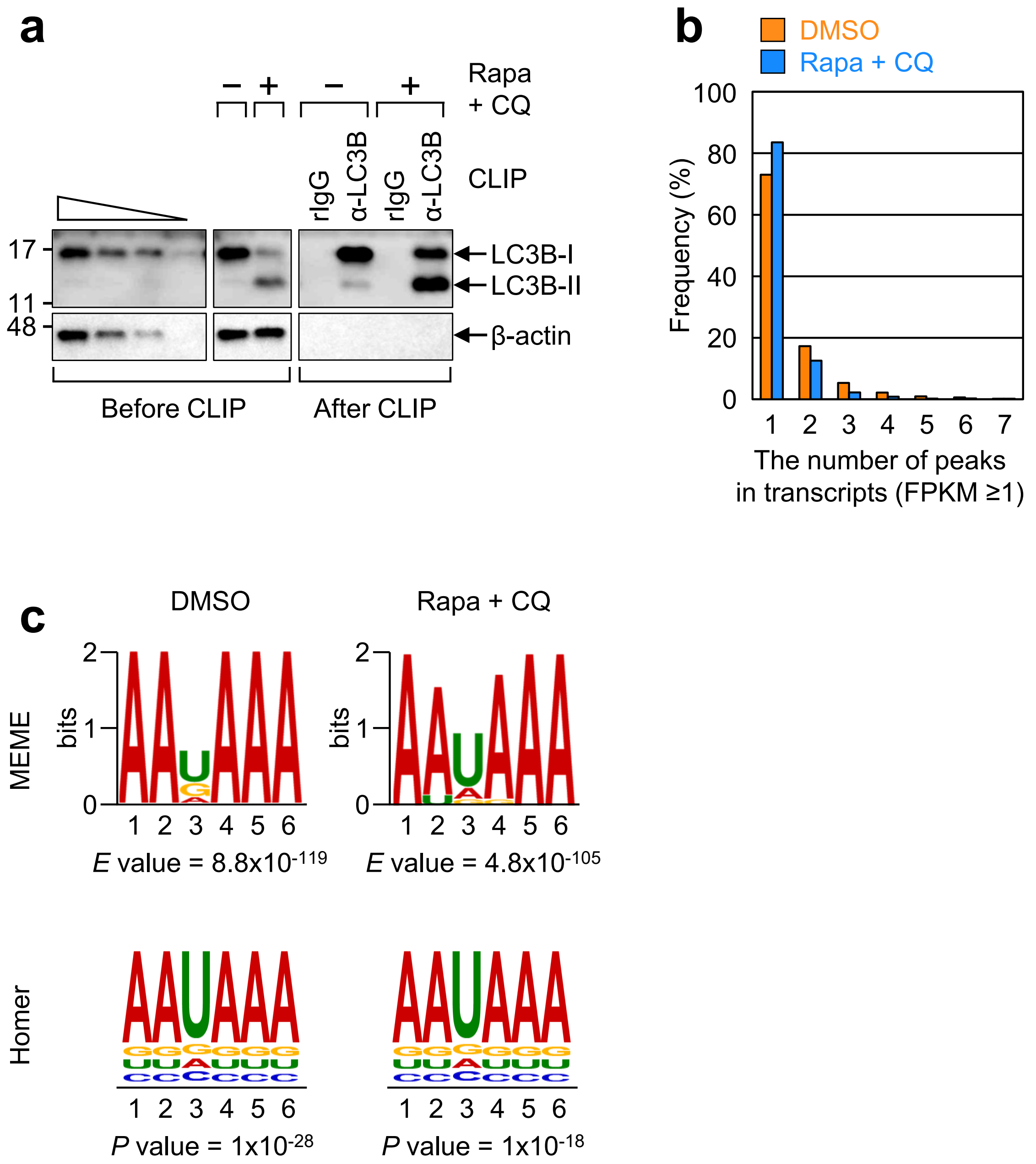

Supplementary Fig. 1. Supporting data for LC3B CLIP-seq. a, Western blotting showing specific IP and efficient LC3B conversion during CLIP-seq experiments in Fig. 1a–d. Protein samples before or after CLIP experiments were analyzed using Western blotting. **b**, The number of the LC3B CLIP peaks in transcripts. **c**, Consensus motif for LC3B binding. As performed for Fig. 1b, except that all LC3B peaks present throughout mRNAs were analyzed. The E-value (upper) estimates the expected number of motifs with a similarly sized set of random sequences using log likelihood ratio. The P values (lower) were calculated using cumulative binomial distributions.

Figure S2

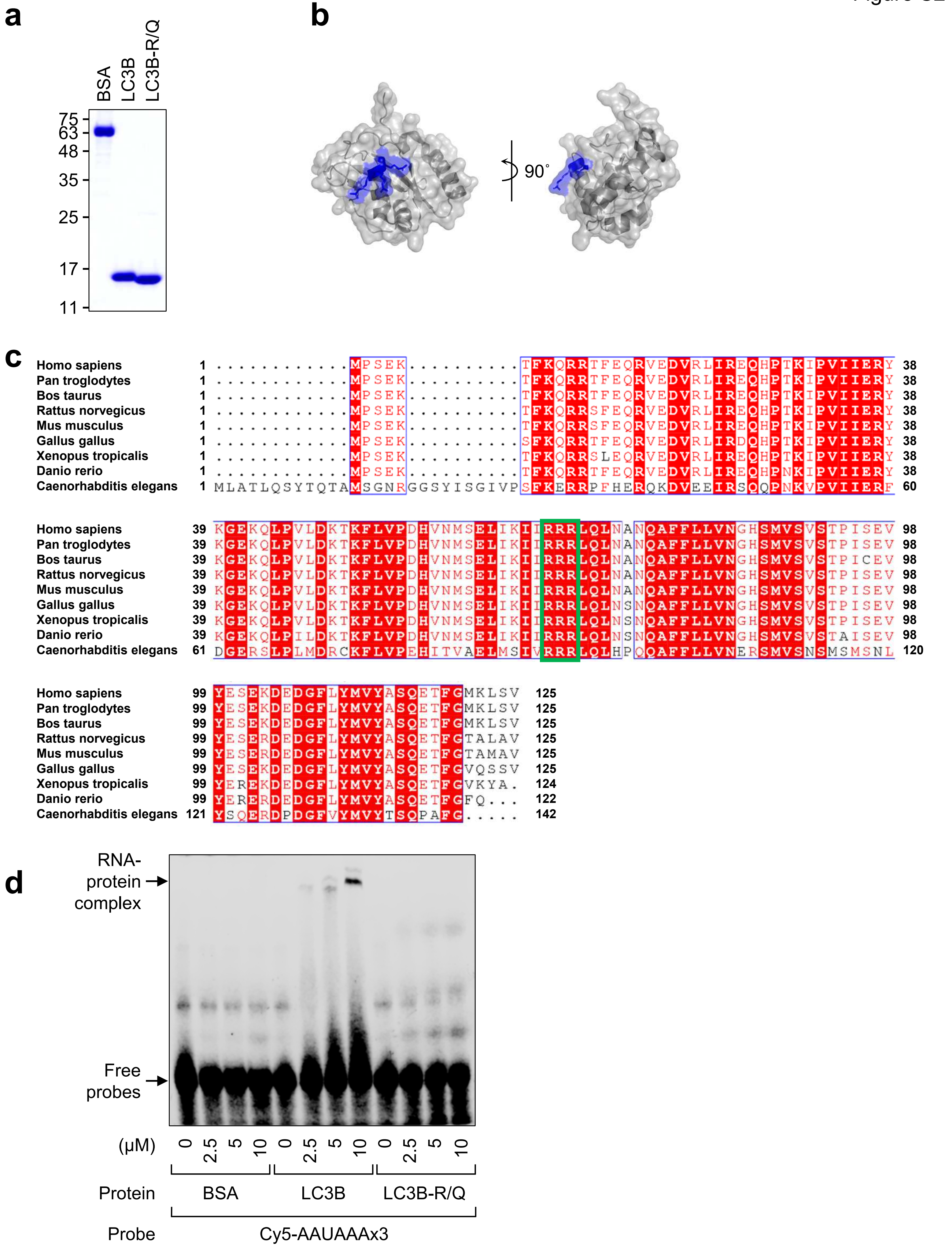
a
BSA
LC3B
LC3B-R/Q
75
63
48
35
25
17
11
b
90˚
c
Homo sapiens 1 ....MPSEK...........TFKQRRTFEQRVEDVRLIREQHPTKIPVIIERY 38
Pan troglodytes 1 ....MPSEK...........TFKQRRTFEQRVEDVRLIREQHPTKIPVIIERY 38
Bos taurus 1 ....MPSEK...........TFKQRRTFEQRVEDVRLIREQHPTKIPVIIERY 38
Rattus norvegicus 1 ....MPSEK...........TFKQRRSFEQRVEDVRLIREQHPTKIPVIIERY 38
Mus musculus 1 ....MPSEK...........TFKQRRSFEQRVEDVRLIREQHPTKIPVIIERY 38
Gallus gallus 1 ....MPSEK...........SFKQRRTFEQRVEDVRLIRDQHPTKIPVIIERY 38
Xenopus tropicalis 1 ....MPSEK...........TFKQRRSLEQRVEDVRLIREQHPTKIPVIIERY 38
Danio rerio 1 ....MPSEK...........TFKQRRTFEQRVEDVRLIREQHPNKIPVIIERY 38
Caenorhabditis elegans 1 MLATLQSYTQTAMSGNRGGSYISGIVPSFKERRPFHERQKDVEEIRSQQPNKVPVIIERF 60
Homo sapiens 39 KGEKQLPVLDKTKFLVPDHVNMSELIKIIRRRLQLNANQAFFLLVNGHSMVSVSTPISEV 98
Pan troglodytes 39 KGEKQLPVLDKTKFLVPDHVNMSELIKIIRRRLQLNANQAFFLLVNGHSMVSVSTPISEV 98
Bos taurus 39 KGEKQLPVLDKTKFLVPDHVNMSELIKIIRRRLQLNANQAFFLLVNGHSMVSVSTPICEV 98
Rattus norvegicus 39 KGEKQLPVLDKTKFLVPDHVNMSELIKIIRRRLQLNANQAFFLLVNGHSMVSVSTPISEV 98
Mus musculus 39 KGEKQLPVLDKTKFLVPDHVNMSELIKIIRRRLQLNANQAFFLLVNGHSMVSVSTPISEV 98
Gallus gallus 39 KGEKQLPVLDKTKFLVPDHVNMSELIKIIRRRLQLNANQAFFLLVNGHSMVSVSTPISEV 98
Xenopus tropicalis 39 KGEKQLPVLDKTKFLVPDHVNMSELIKIIRRRLQLNSNQAFFLLVNGHSMVSVSTPISEV 98
Danio rerio 39 KGEKQLPILDKTKFLVPDHVNMSELIKIIRRRLQLNSNQAFFLLVNGHSMVSVSTAISEV 98
Caenorhabditis elegans 61 DGERSLPLMDRCKFLVPEHITVAELMSIVRRRLQLHPQQAFFLLVNERSMVSNSMSMSNL 120
Homo sapiens 99 YESEKDEDGFLYMVYASQETFGMKLSV 125
Pan troglodytes 99 YESEKDEDGFLYMVYASQETFGMKLSV 125
Bos taurus 99 YESEKDEDGFLYMVYASQETFGMKLSV 125
Rattus norvegicus 99 YESERDEDGFLYMVYASQETFGTALAV 125
Mus musculus 99 YESERDEDGFLYMVYASQETFGTAMAV 125
Gallus gallus 99 YESEKDEDGFLYMVYASQETFGVQSSV 125
Xenopus tropicalis 99 YEREKDEDGFLYMVYASQETFGVKYA. 124
Danio rerio 99 YERERDEDGFLYMVYASQETFGFQ... 122
Caenorhabditis elegans 121 YSQERDPDGFVYMVYTSQPAFG..... 142
d
RNA-protein complex
Free probes
(μM) 0 2.5 5 10 0 2.5 5 10 0 2.5 5 10
Protein BSA LC3B LC3B-R/Q
Probe Cy5-AAUAAAx3

Supplementary Fig. 2. Supporting data for LC3B-R/Q variant. **a**, Coomassie blue staining of purified recombinant proteins showing protein integrity. **b**, Structure of human LC3B (PDB: 1V39) shown as ribbon with transparent surface. The hydrophobic property is presented in the molecular surface of LC3B. Three arginine (R) residues at positions of 68, 69, and 70 are specified by blue and the other structures are colored with gray. **c**, Amino acid alignment of LC3B among different species. Three arginine (R) residues at positions of 68, 69, and 70 are indicated by thick green line. **d**, EMSA analysis using *in vitro*-synthesized Cy5-labeled triple repeats (×3) of AAUAAA and either purified recombinant LC3B-WT or LC3B-R/Q. Representative data obtained from two independently performed biological replicates (n = 2) are shown. Source data are provided as a Source Data file.

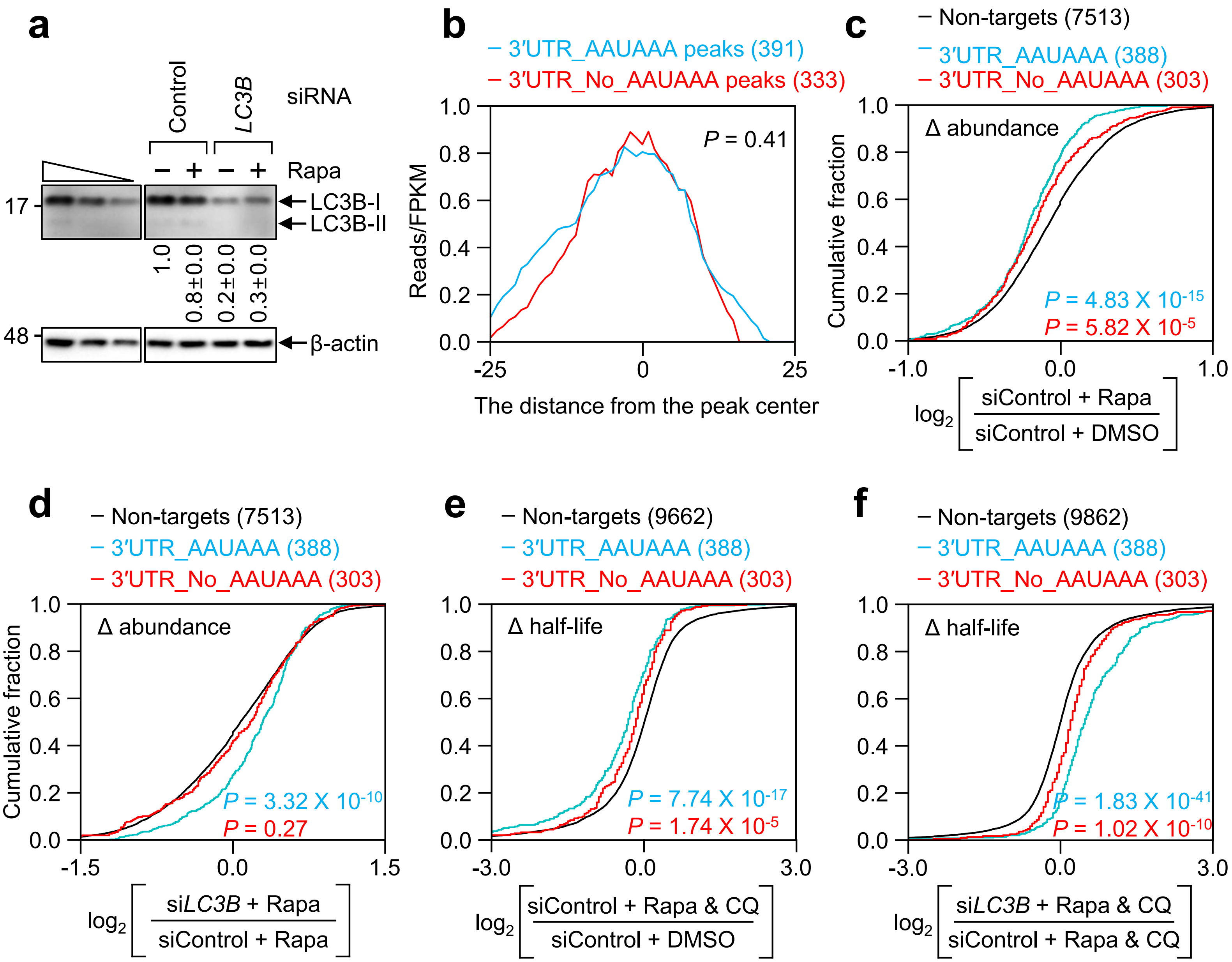

Supplementary Fig. 3. Binding of LC3B to 3’UTR of target mRNAs triggers efficient mRNA degradation. a, Western blotting showing specific downregulation of endogenous LC3B in Fig. 2. **b**, Reads / FPKM values of LC3B peaks located at 3′UTR. The LC3B peak in the 3′UTR were categorized into two groups: 3′UTR_AAUAAA peaks group (which harbors the LC3B peaks at the consensus AAUAAA motif) and 3′UTR_No_AAUAAA peak group (which harbors the LC3B peaks elsewhere rather than AAUAAA). **c, d**, CDF plots for the relative changes in the abundance of mRNAs harboring the AAUAAA motif upon Rapa treatment in the undepleted cells (**c**) or upon LC3B downregulation in the Rapa-treated cells (**d**). The mRNAs harboring the LC3B peak in the 3′UTR (3′UTR group in Fig. 2b, c) were categorized into two groups: the 3′UTR_AAUAAA group (which harbors the LC3B peaks at the consensus AAUAAA motif) and the 3′UTR_No_AAUAAA group (which harbors the LC3B peaks elsewhere rather than AAUAAA). **e, f**, CDF plots for the relative change in the half-life of mRNAs belonging to the 3′UTR_AAUAAA or 3′UTR_No_AAUAAA group upon Rapa + CQ treatment in the undepleted cells (**e**) or upon LC3B downregulation in the cells treated with Rapa + CQ (**f**). P values were calculated using the two-tailed Mann–Whitney *U* test. Source data are provided as a Source Data file.

Figure S4

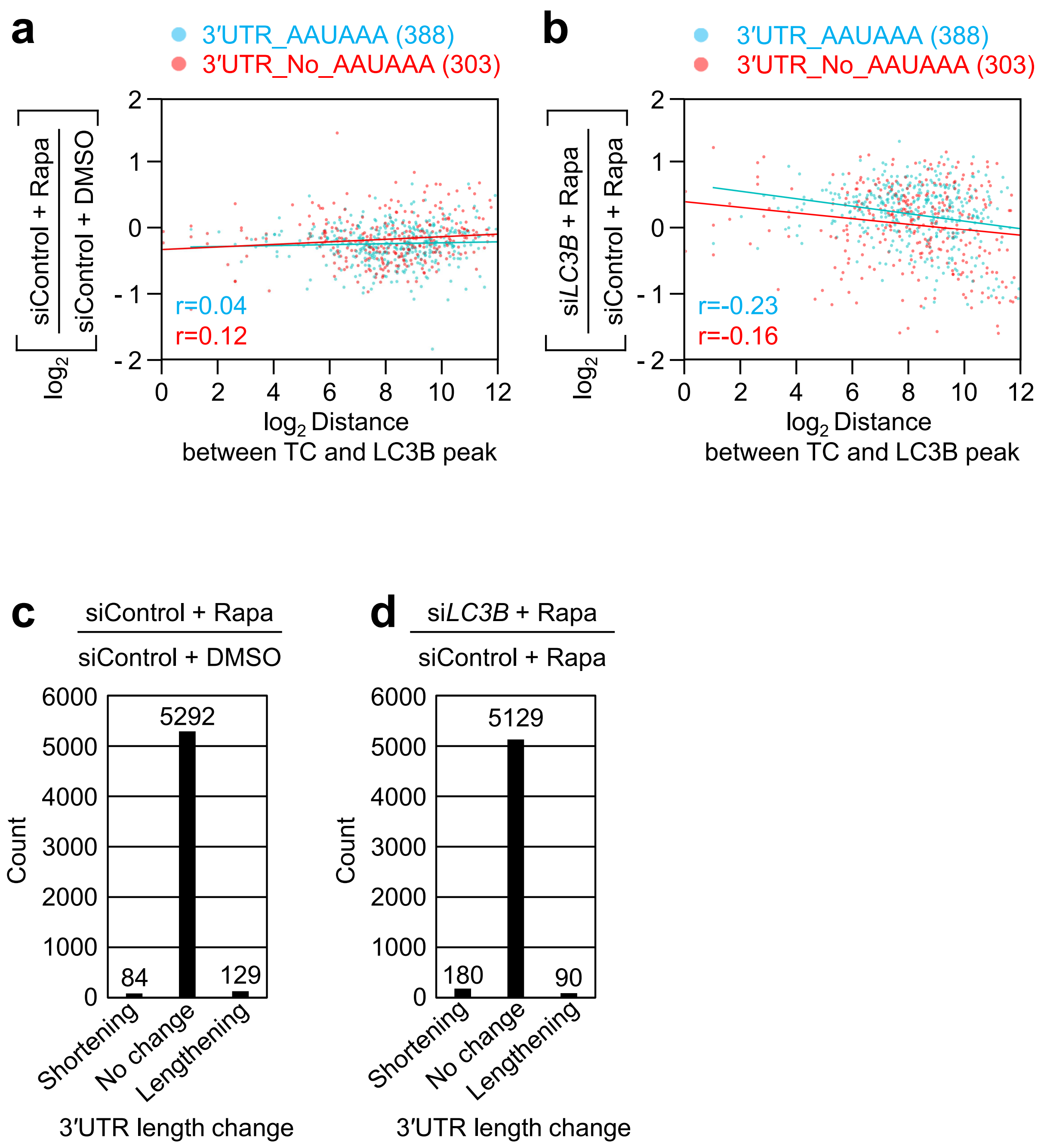

Supplementary Fig. 4. Binding of LC3B to target mRNAs marginally affects alternative splicing. a, Scatter plot of the change in mRNA abundance upon Rapa treatment and the distance between the translation termination codon (TC) and the LC3B peak. The x-axis presents the distance between a TC and LC3B peak. The y-axis presents the relative ratio of mRNA abundance in the Rapa-treated cells to that in the DMSO-treated cells. The mRNAs belonging to the 3′UTR_AAUAAA group and the 3′UTR_No_AAUAAA group were plotted and indicated by blue and red dots, respectively. The Pearson′s correlation coefficient (r) was calculated to determine a significant correlation. **b**, Scatter plot of the change in mRNA abundance upon LC3B downregulation and the distance between a TC and the LC3B peak. As performed in panel **a**, except that the y-axis presents the relative ratio of mRNA abundance in the Rapa-treated and LC3B-depleted cells to that in the Rapa-treated and undepleted cells. **c**, Effect of Rapa treatment on the change in the length of 3′UTR. The mRNAs with FPKM ≥ 1 were used for calculations. **d,** Effect of LC3B downregulation in the cells treated with Rapa on the change in the length of 3′UTR. The mRNAs with FPKM ≥ 1 were used for calculations.

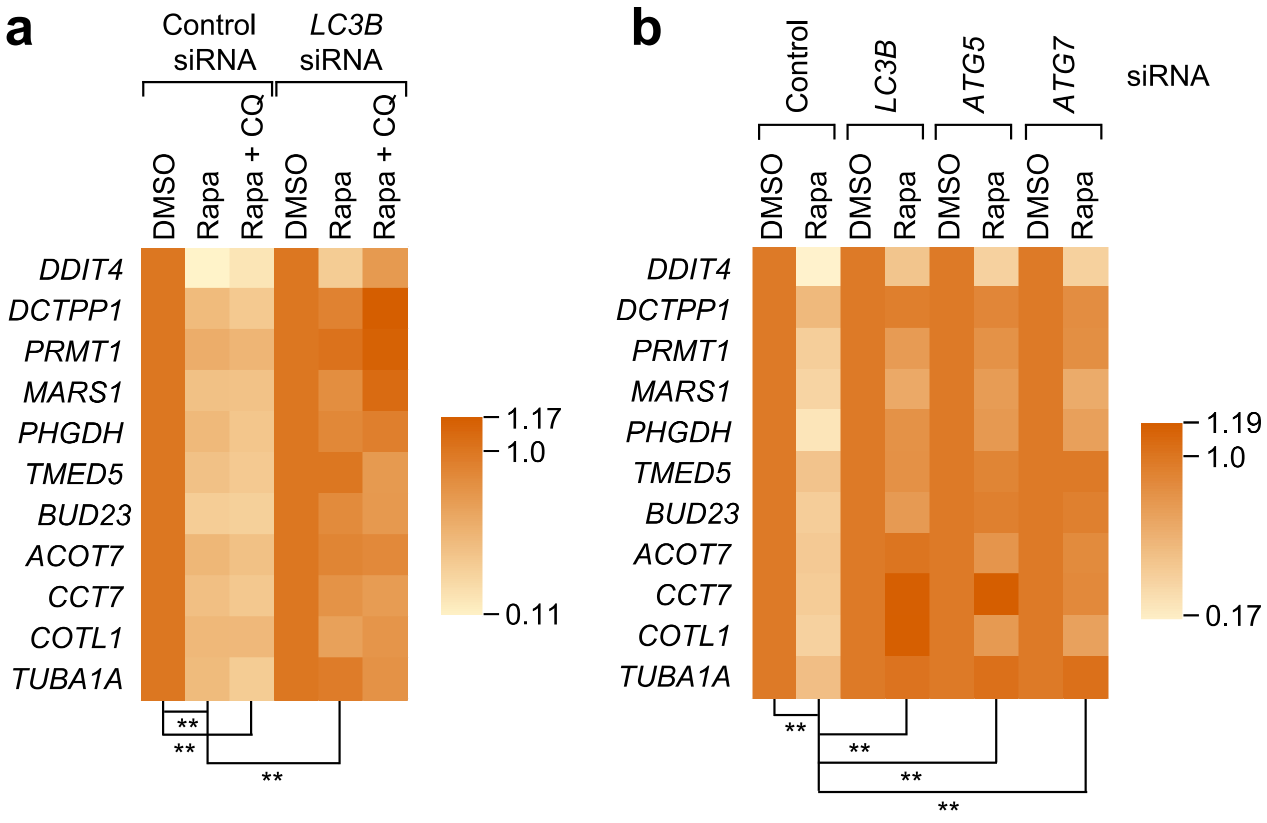

Supplementary Fig. 5. Validation of potential LMD substrates. a, Heat map of potential LMD targets. HEK293T cells either undepleted or depleted of endogenous LC3B were treated with DMSO, Rapa, or Rapa + CQ. n = 5. The two-tailed Wilcoxon signed-rank test was conducted to calculate the P values. **, P < 0.01 (The exact P values are provided in Source Data file). **b**, Heat map of potential LMD targets upon downregulation of LC3B, ATG5, or ATG7. HEK293T cells depleted of endogenous LC3B, ATG5, or ATG7 were treated with either DMSO or Rapa. The amounts of LMD substrates were normalized to the amount of *GAPDH* mRNA. The normalized levels in the cells treated with DMSO were arbitrarily set to 1.0; n = 3; The two-tailed Wilcoxon signed–ranked test was conducted to calculate the P values. **, P < 0.01 (The exact P values are provided in Source Data file). Source data are provided as a Source Data file.

Figure S6

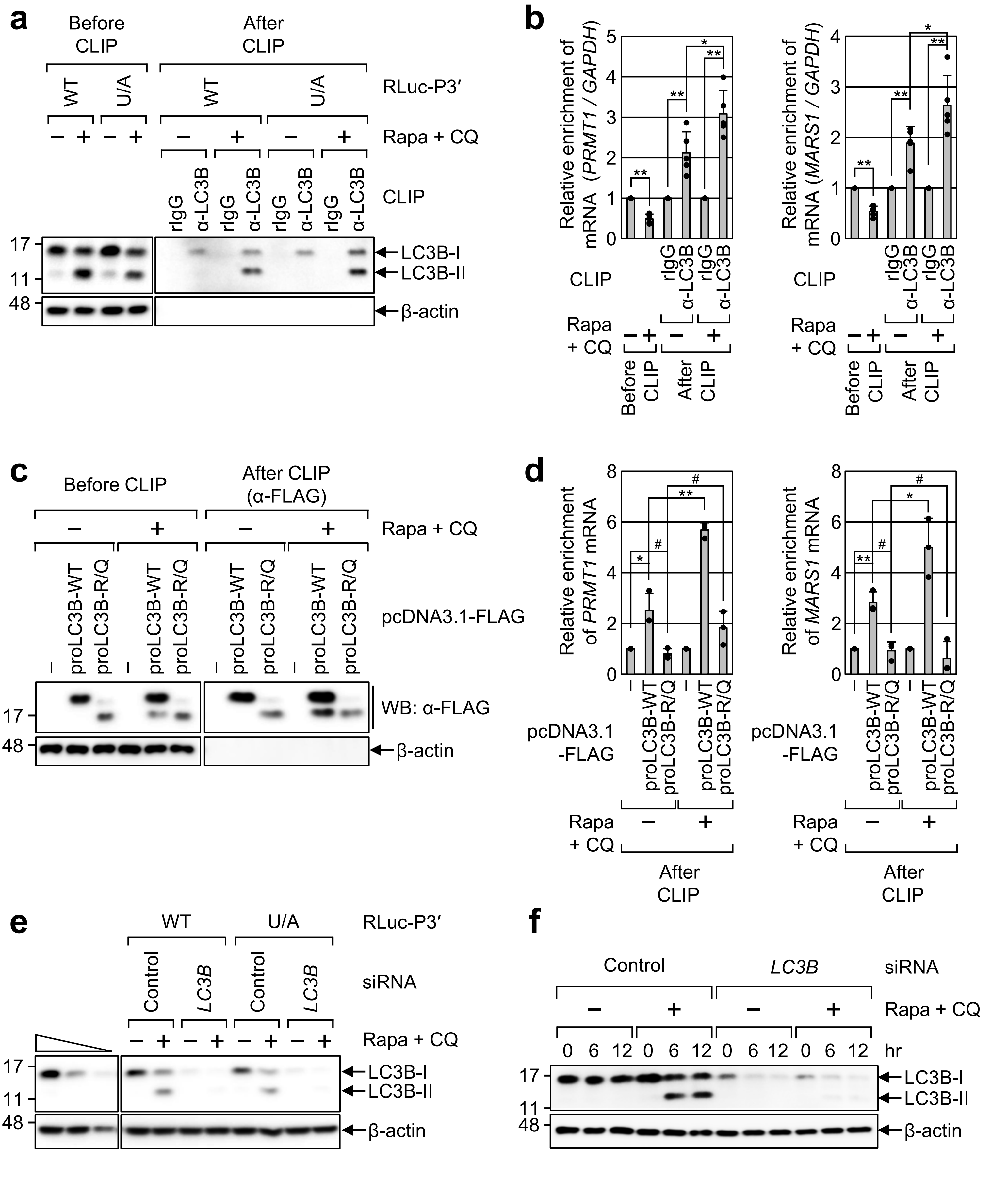

a
Before CLIP
After CLIP
WT
U/A
RLuc-P3′
Rapa + CQ
CLIP
rIgG
α-LC3B
LC3B-I
LC3B-II
β-actin
17
11
48
b
Relative enrichment of mRNA (PRMT1 / GAPDH)
Relative enrichment of mRNA (MARS1 / GAPDH)
CLIP
Rapa + CQ
Before CLIP
After CLIP
c
Before CLIP
After CLIP (α-FLAG)
pcDNA3.1-FLAG
proLC3B-WT
proLC3B-R/Q
WB: α-FLAG
d
Relative enrichment of PRMT1 mRNA
Relative enrichment of MARS1 mRNA
pcDNA3.1 -FLAG
e
siRNA
Control
LC3B
f
hr

Supplementary Fig. 6. Supporting data for Fig. 3. a, Western blotting showing IP specificity in Fig. 3c. **b**, Relative amounts of coimmunoprecipitated endogenous *PRMT1* mRNA (left) and *MARS1* mRNA (right) in Fig. 3c. n = 5; Data are presented as mean values ± S.D.; P values were analyzed using two-tailed and equal-variance Student's t-test; *, P < 0.05; **, P < 0.01 (The exact P values are provided in Source Data file). **c, d**, *In vivo* CLIP of either proLC3B-WT or proLC3B-R/Q. As performed in Fig. 3c, except that HEK293T cells expressing either proLC3B-WT or proLC3B-R/Q were subjected to *in vivo* CLIP using α-FLAG antibody. The amounts of co-immunoprecipitated endogenous mRNAs were normalized to those of endogenous *GAPDH* mRNAs. Then, the normalized levels obtained in IPs in the untreated cells expressing only FLAG were arbitrarily set to 1.0; n = 3; Data are presented as mean values ± S.D. P values were analyzed using two-tailed and equal-variance Student's *t*-test; #, not significant; *, P < 0.05; **, P < 0.01 (The exact P values are provided in Source Data file). **e**, Western blotting proving specific downregulation of endogenous LC3B in Fig. 3d. **f**, Western blotting showing specific downregulation of endogenous LC3B in Fig. 3e–g. Source data are provided as a Source Data file.

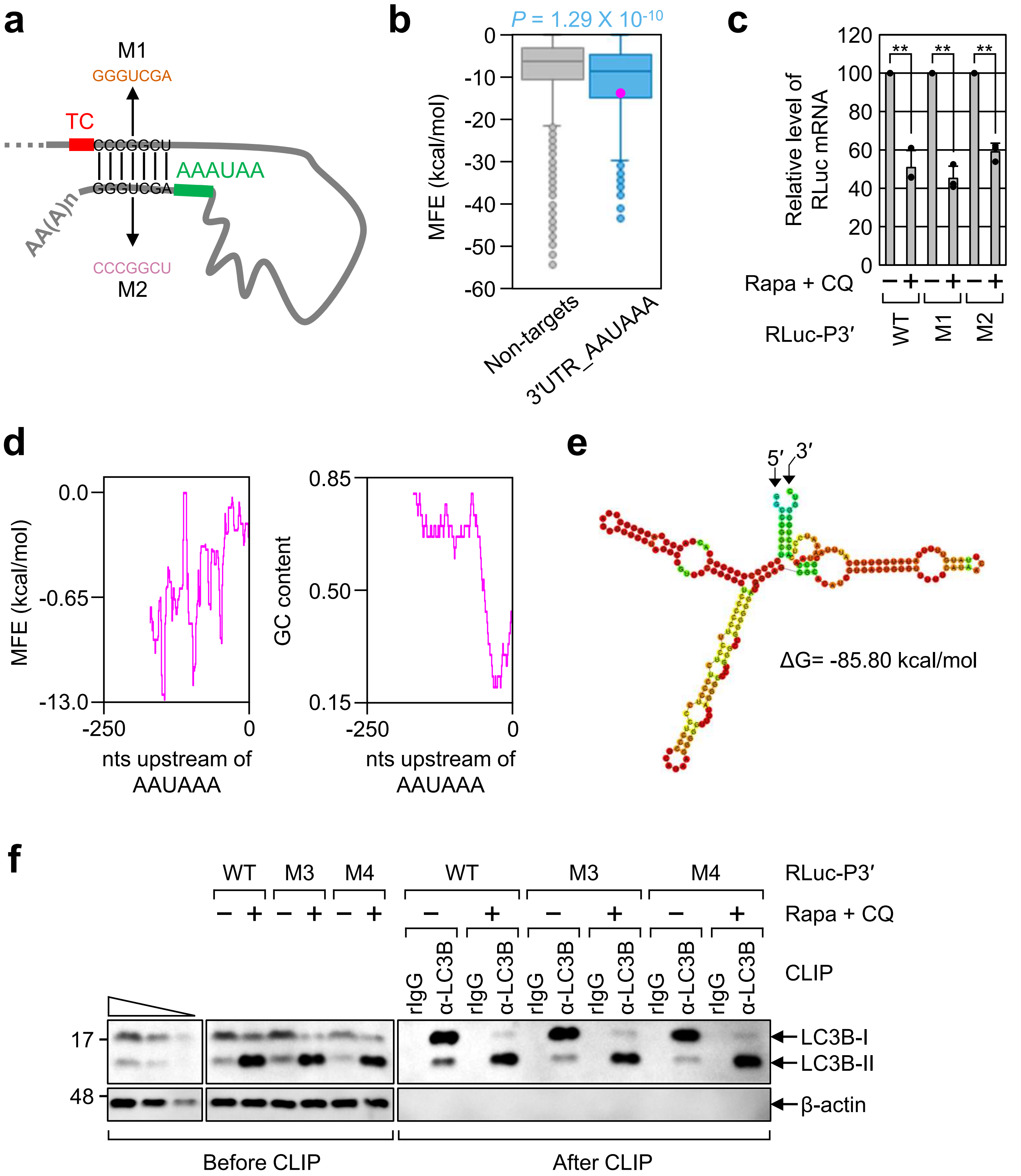
a
M1
GGGUCGA
TC
CCCGGCU
AAAUAA
GGGUCGA
AA(A)n
CCCGGCU
M2
b
P = 1.29 X 10-10
MFE (kcal/mol)
0
-10
-20
-30
-40
-50
-60
Non-targets
3′UTR_AAUAAA
c
Relative level of RLuc mRNA
120
100
80
60
40
20
0
**
Rapa + CQ
RLuc-P3′
WT
M1
M2
d
MFE (kcal/mol)
0.0
-0.65
-13.0
-250
0
nts upstream of AAUAAA
GC content
0.85
0.50
0.15
e
5′
3′
ΔG= -85.80 kcal/mol
f
WT
M3
M4
RLuc-P3′
Rapa + CQ
CLIP
rIgG
α-LC3B
17
48
LC3B-I
LC3B-II
β-actin
Before CLIP
After CLIP

Supplementary Fig. 7. Supporting data for Fig. 4. a, Schematic diagram for a long-range looping in the *PRMT1* 3′UTR. Seven nucleotide substitutions immediately downstream of the translation termination codon (TC; RLuc-P3′-M1 mRNA) and seven nucleotide substitutions immediately downstream of the AAUAAA motif (RLuc-P3′-M2 mRNA) are depicted in the diagram. **b**, Comparison of the minimum free energy (MFE) for a long-range interaction. The MFE values of the 3′UTR_AAUAAA group and non-targets were calculated. The *PRMT1* mRNA is indicated by the red dot. P values were calculated using the two-tailed Kolmogorov–Smirnov test. The number of genes per group: n = 7,513 for non-targets and n = 388 for 3′UTR_AAUAAA group. Box-whiskers show maximum, third quartile to first quartile, median, and minimum. **c**, Relative abundance of the RLuc-P3′ reporter mRNAs. HEK293T cells were transiently transfected with a plasmid expressing the RLuc-P3′ reporter mRNA (WT, M1, or M2) and a plasmid expressing the FLuc reporter mRNA. The cells were either treated or not treated with Rapa + CQ 12 h before cell harvest. The amounts of RLuc mRNAs were normalized to those of FLuc mRNAs. Then, the normalized levels in the untreated cells were arbitrarily set to 100%; n = 3; Data are presented as mean values ± S.D. P values were analyzed using two-tailed and equal-variance Student's *t*-test; **, P < 0.01 (The exact P values are provided in Source Data file). **d**, MFE values (left) and GC content (right) across the nucleotide positions upstream of the AAUAAA motif at the 3′UTR of *PRMT1* mRNA. **e**, Predicted secondary structure of *PRMT1* 3′UTR. ΔG = -85.80 kcal/mol. **f**, Western blotting showing the efficient conversion of LC3B and specificity of IPs in Fig. 4d. Source data are provided as a Source Data file.

Figure S8

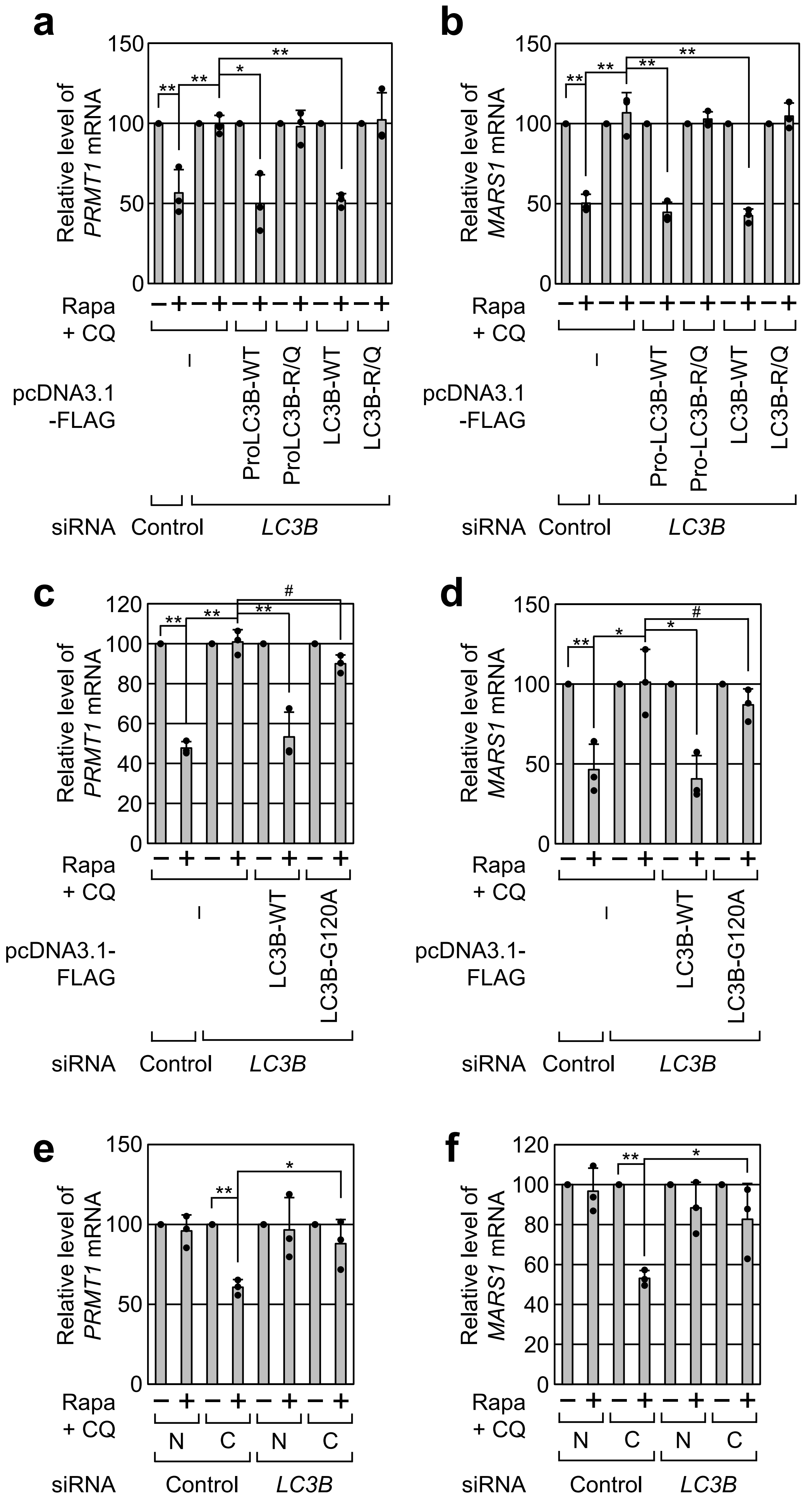
a
b
c
d
e
f
Relative level of PRMT1 mRNA
Relative level of MARS1 mRNA
150
120
100
80
60
50
40
20
0
**
*
#
Rapa + CQ
pcDNA3.1 -FLAG
pcDNA3.1- FLAG
ProLC3B-WT
ProLC3B-R/Q
Pro-LC3B-WT
Pro-LC3B-R/Q
LC3B-WT
LC3B-R/Q
LC3B-G120A
N
C
siRNA
Control
LC3B

Supplementary Fig. 8. Efficient LMD depends on RNA-binding ability and PE conjugation of LC3B. a, b, Complementation experiments using LC3B variants. As performed in Fig. 5b, c, except that the abundance of endogenous LMD substrates *PRMT1* mRNA (**a**) and *MARS1* mRNA (**b**) were measured. n = 3; *, $P < 0.05$; **, $P < 0.01$. **c, d**, Complementation experiments using LC3B-WT and LC3B-G120A. As performed in Fig. 5d, e, except that the abundance of endogenous LMD substrates *PRMT1* mRNA (**c**) and *MARS1* mRNA (**d**) were measured. n = 3; #, not significant; *, $P < 0.05$; **, $P < 0.01$. **e, f**, Efficiency of LMD in the nuclear and cytoplasmic extracts. As performed in Fig. 5f, g, except that the abundance of endogenous LMD substrates *PRMT1* mRNA (**e**) and *MARS1* mRNA (**f**) were measured. n = 3; *, $P < 0.05$; **, $P < 0.01$. In all panels, data are presented as mean values ± S.D.; P values were analyzed using two-tailed and equal-variance Student's *t*-test. The exact P values are provided in Source Data file.

Figure S9

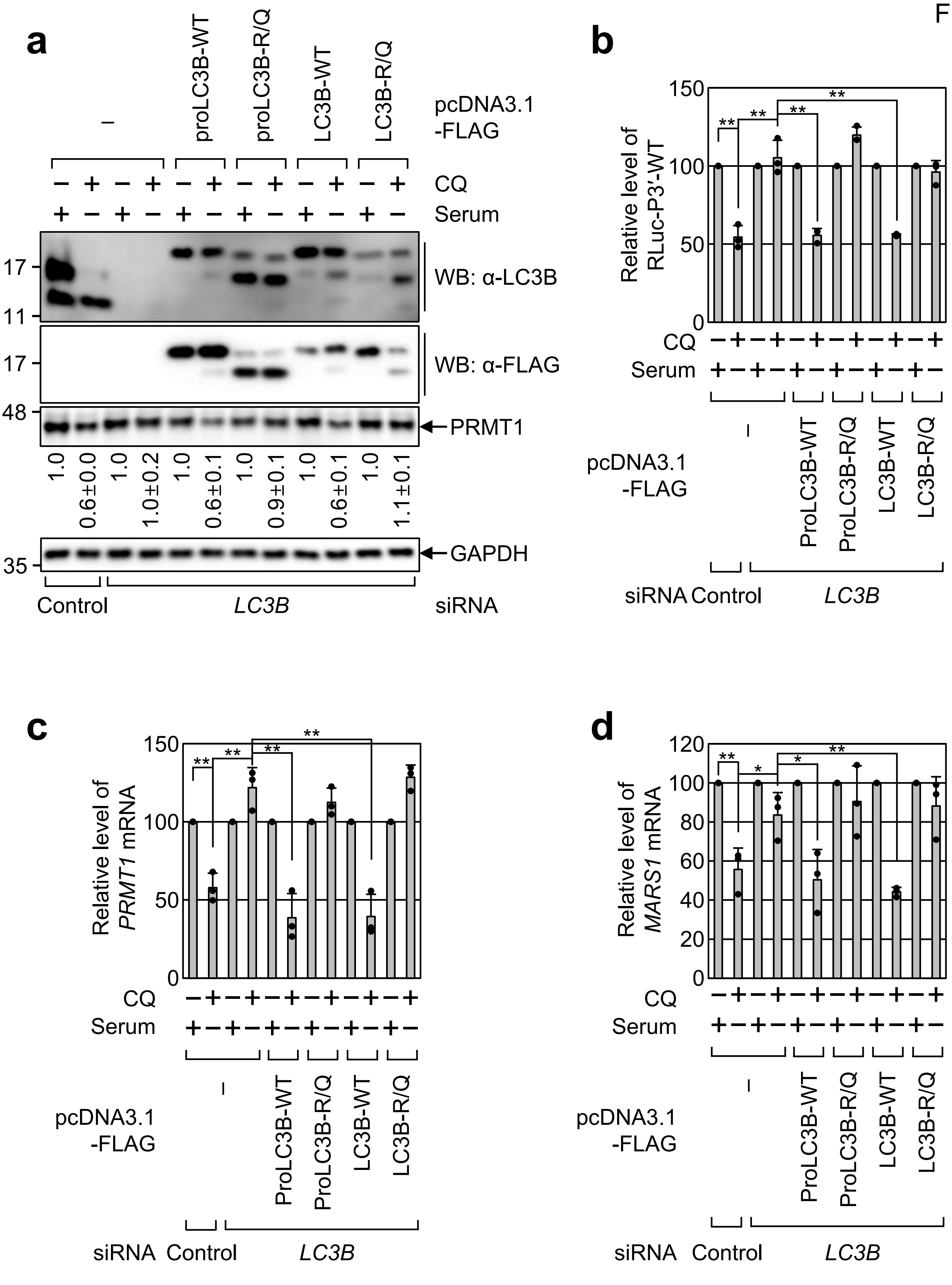

Supplementary Fig. 9. Serum starvation induces LMD. As performed in Fig. 5b, c, except that autophagy was induced by serum starvation for 12 h. **a**, Western blotting showing specific downregulation of endogenous LC3B and proper expression of FLAG-LC3B or its variant. **b–d**, Effect of LC3B-WT or its variant on LMD of RLuc-P3′-WT reporter mRNA (**b**), endogenous *PRMT1* mRNA (**c**), and endogenous *MARS1* mRNA (**d**); n = 3; Data are presented as mean values ± S.D. P values were analyzed using two-tailed and equal-variance Student's *t*-test; *, $P < 0.05$; **, $P < 0.01$ (The exact P values are provided in Source Data file). Source data are provided as a Source Data file.

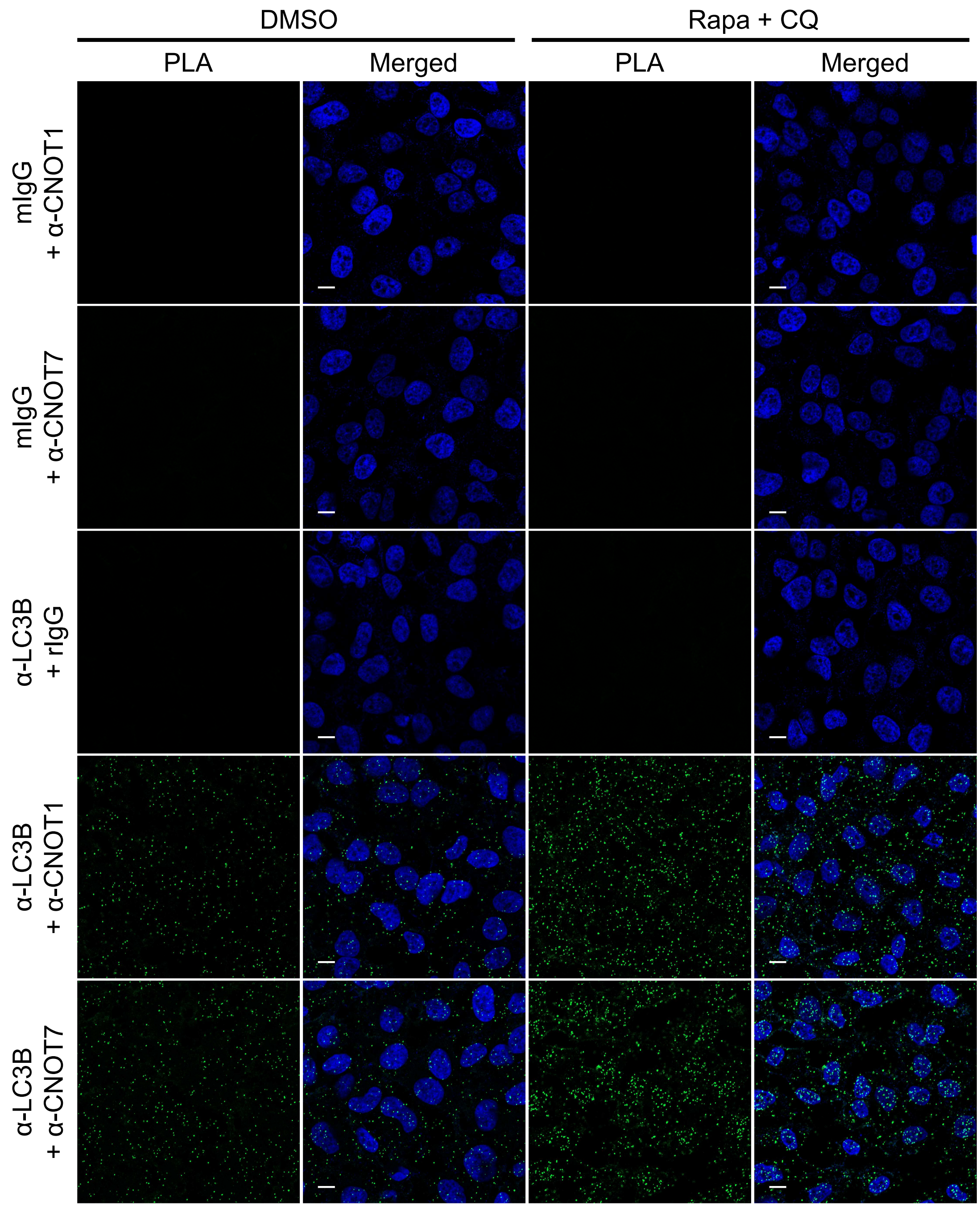

Supplementary Fig. 10. Proximity ligation assay (PLA) between endogenous LC3B and either CNOT1 or CNOT7. The PLA experiments were performed as described in Fig. 6d. Scale bar = 10 μm.

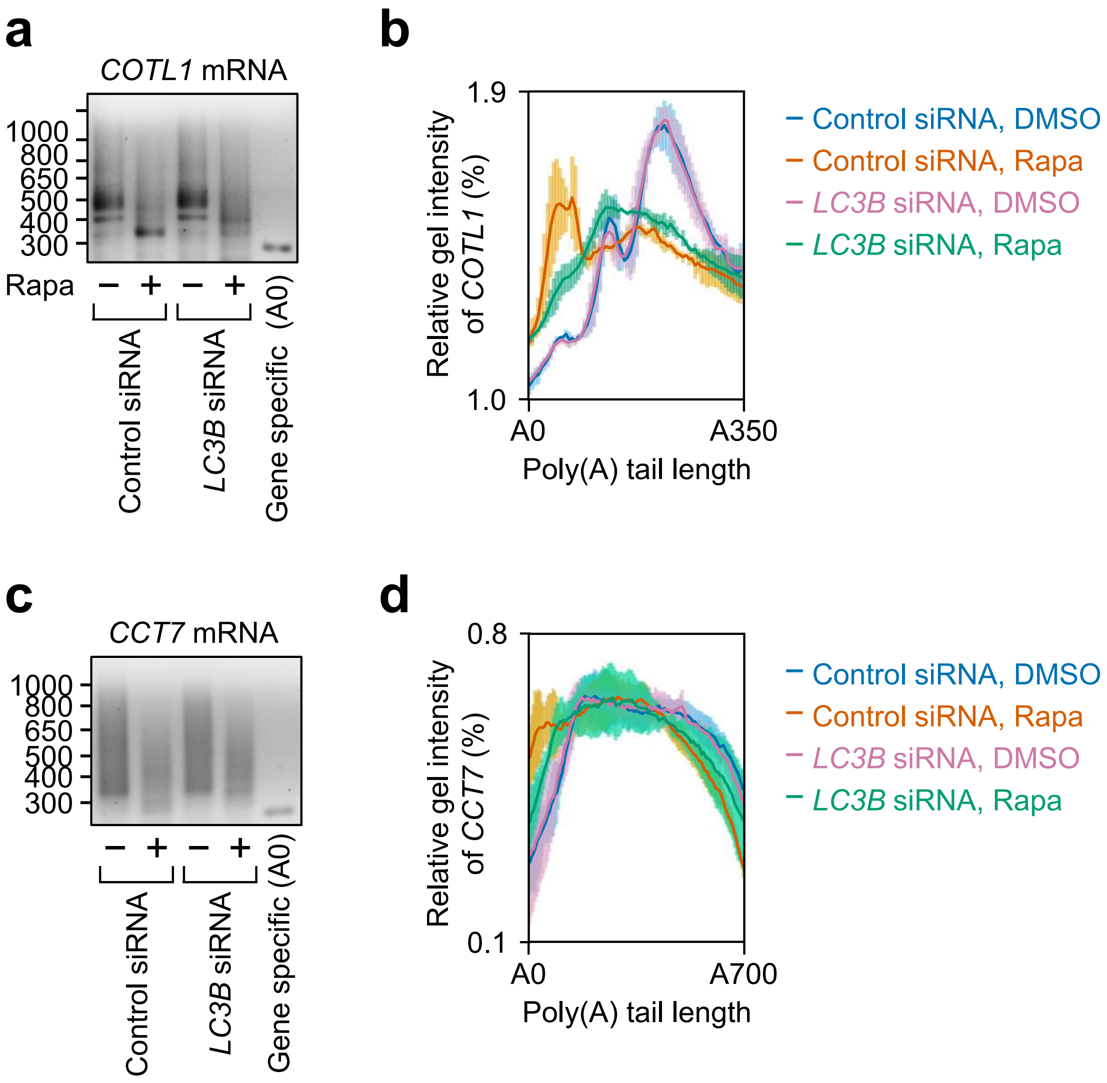

Supplementary Fig. 11. LMD involves poly(A) shortening. HEK293T cells were either not treated or treated with Rapa for 12 h. Total cell RNA was purified, and the changes in the length of poly(A) tail of LMD substrates were analyzed as described in the Materials and Methods section. Gene specific A(0) indicates PCR products lacking the poly(A) tail of endogenous LMD substrates under our conditions. **a, c**, Agarose gel images showing the changes in the length of poly(A) tail of COTL1 mRNA (**a**) and CCT7 mRNA (**c**). **b, d**, Quantitation of agarose gel images corresponding to poly(A) tails in panels a (**b**) and c (**d**). Mean values and standard deviations of the relative intensities obtained from three biological replicates are shown as solid lines and semi-transparent lines, respectively. Source data are provided as a Source Data file.

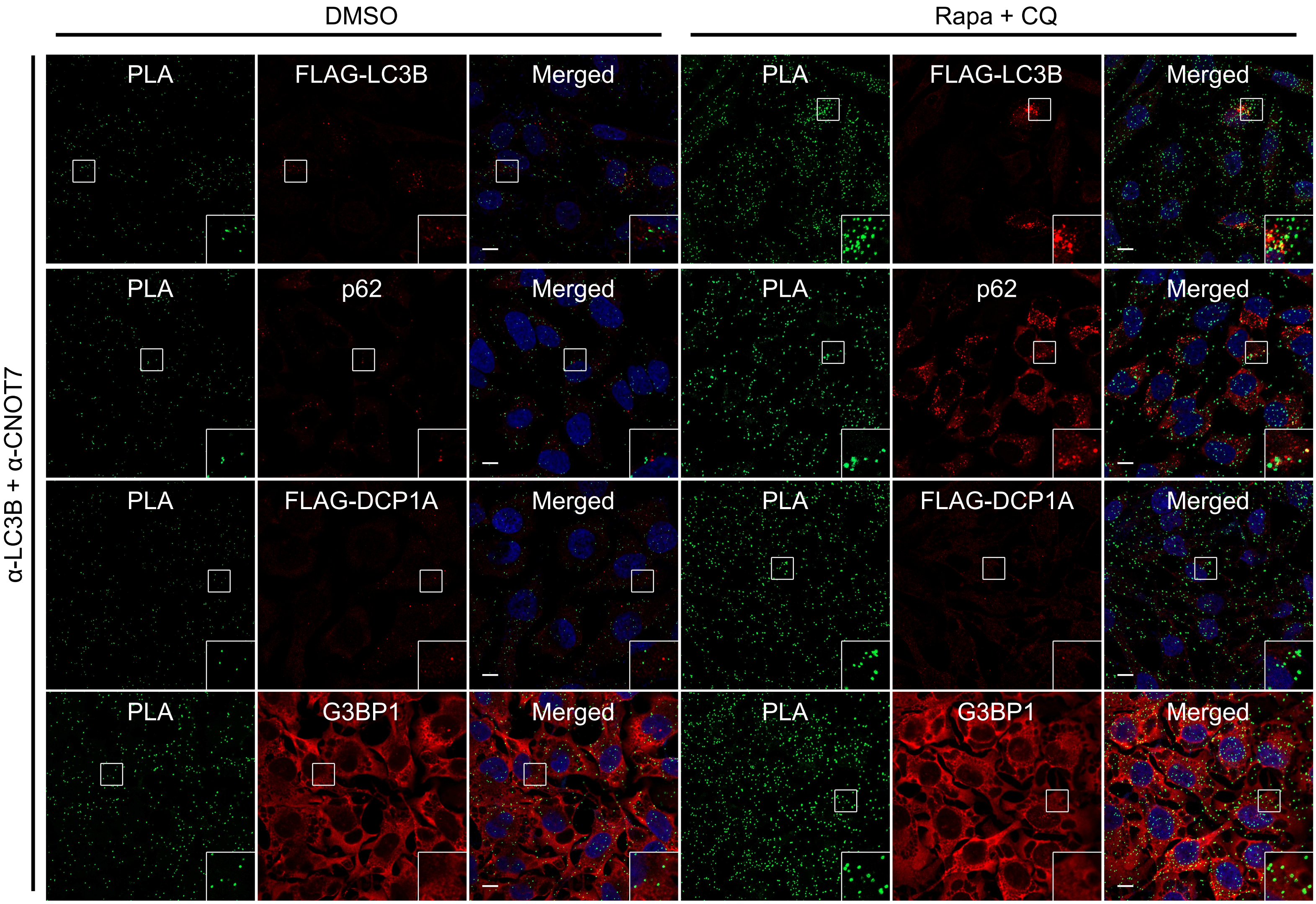

Supplementary Fig. 12. Intracellular distributions of PLA spots involving endogenous LC3B and CNOT7. As performed in Fig. 7a, except that the specific interaction between endogenous LC3B and CNOT7 was determined using PLA experiments. n = 3; Scale bar = 10 µm.

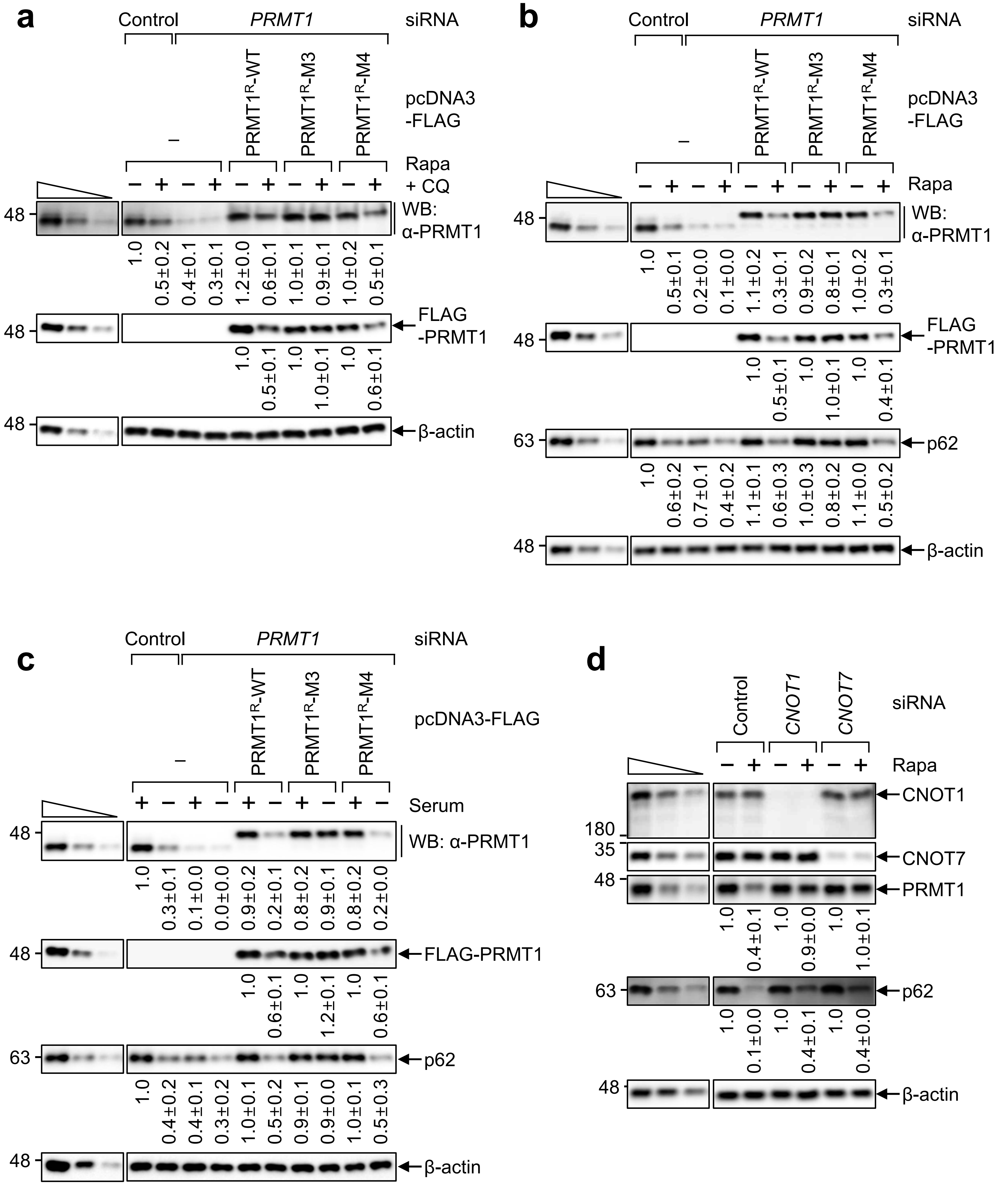
a
Control
PRMT1
siRNA
pcDNA3
-FLAG
PRMT1R-WT
PRMT1R-M3
PRMT1R-M4
Rapa
+ CQ
WB:
α-PRMT1
FLAG
-PRMT1
β-actin
48
b
Control
PRMT1
siRNA
pcDNA3
-FLAG
Rapa
WB:
α-PRMT1
FLAG
-PRMT1
p62
β-actin
63
c
Control
PRMT1
siRNA
pcDNA3-FLAG
Serum
WB: α-PRMT1
FLAG-PRMT1
p62
β-actin
d
Control
CNOT1
CNOT7
siRNA
Rapa
CNOT1
CNOT7
PRMT1
p62
β-actin
180
35

Supplementary Fig. 13. Supporting data for Fig. 8. a, Western blotting of PRMT1 in Fig. 8b–d. **b**, Complementation experiment using the FLAG-PRMT1^{R} reporter mRNAs under Rapa-treated conditions. As performed in Fig. 8b–d, except that the cells were treated with Rapa for 12 h; n = 3. **c**, Complementation experiment using the FLAG-PRMT1^{R} reporter mRNAs under serum-depleted conditions. As performed in Fig. 8b–d, except that the cells were either not treated or treated with serum starvation for 12 h; n = 3. **d**, Effect on downregulation of either CNOT1 or CNOT7. HEK293T cells depleted of either CNOT1 or CNOT7 were treated with Rapa for 12 h; n = 2. Source data are provided as a Source Data file.

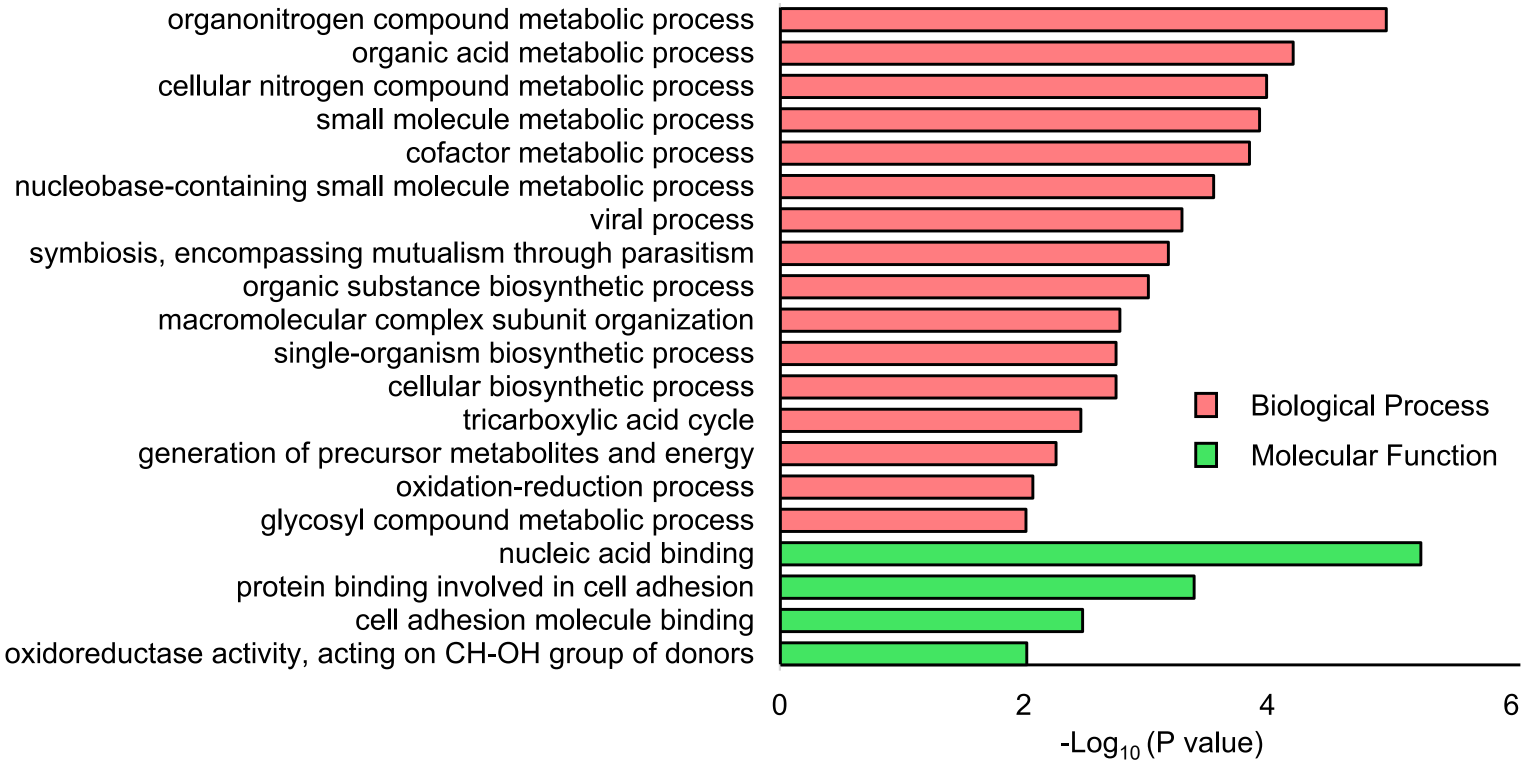

Supplementary Fig. 14. Gene ontology analyses of putative LMD substrates. We analyzed a list of genes, which harbored LC3B peaks in the AAUAAA motif and showed a decrease in abundance upon Rapa treatment at least 1.3-fold and an increase in abundance upon LC3B downregulation at least 1.3-fold.
