# Supplementary material for: LC3B is an RNA-binding protein to trigger rapid mRNA degradation during autophagy
Source: Nat Commun. 2022 Mar 17;13:1436. doi: 10.1038/s41467-022-29139-1 (PMC8931120; doi:10.1038/s41467-022-29139-1)
Supplement: Supplementary file 3 — Description of Additional Supplementary Files [file 41467_2022_29139_MOESM3_ESM.pdf]

Description of Additional Supplementary Files

File Name: Supplementary Data 1
Description: The numbers of reads obtained from stepwise processing. a, LC3B CLIP-seq. b, mRNA-seq for input RNA in the CLIP-seq experiments. c, mRNA-seq to compare mRNA abundance. d, mRNA-seq for half-life measurements.

File Name: Supplementary Data 2
Description: List of mRNAs harboring LC3B peaks at the consensus AAUAAA motif.

File Name: Supplementary Data 3
Description: Sequence information on siRNAs (a), oligonucleotides for qRT-PCRs (b), and poly(A) tail-length assay (c).
