# Supplementary material for: LC3B is an RNA-binding protein to trigger rapid mRNA degradation during autophagy
Source: Nat Commun. 2022 Mar 17;13:1436. doi: 10.1038/s41467-022-29139-1 (PMC8931120; doi:10.1038/s41467-022-29139-1)
Supplement: Supplementary file 7 — Reporting Summary [file 41467_2022_29139_MOESM7_ESM.pdf]

nature research

Corresponding author(s): Kim, Yoon Ki

Last updated by author(s): Feb 7, 2022

Reporting Summary

Nature Research wishes to improve the reproducibility of the work that we publish. This form provides structure for consistency and transparency in reporting. For further information on Nature Research policies, see our Editorial Policies and the Editorial Policy Checklist.

Statistics

For all statistical analyses, confirm that the following items are present in the figure legend, table legend, main text, or Methods section.

n/a	Confirmed	
☐	☒	The exact sample size (*n*) for each experimental group/condition, given as a discrete number and unit of measurement
☐	☒	A statement on whether measurements were taken from distinct samples or whether the same sample was measured repeatedly
☐	☒	The statistical test(s) used AND whether they are one- or two-sided<br>*Only common tests should be described solely by name; describe more complex techniques in the Methods section.*
☐	☒	A description of all covariates tested
☐	☒	A description of any assumptions or corrections, such as tests of normality and adjustment for multiple comparisons
☐	☒	A full description of the statistical parameters including central tendency (e.g. means) or other basic estimates (e.g. regression coefficient) AND variation (e.g. standard deviation) or associated estimates of uncertainty (e.g. confidence intervals)
☐	☒	For null hypothesis testing, the test statistic (e.g. *F*, *t*, *r*) with confidence intervals, effect sizes, degrees of freedom and *P* value noted<br>*Give P values as exact values whenever suitable.*
☒	☐	For Bayesian analysis, information on the choice of priors and Markov chain Monte Carlo settings
☒	☐	For hierarchical and complex designs, identification of the appropriate level for tests and full reporting of outcomes
☐	☒	Estimates of effect sizes (e.g. Cohen's *d*, Pearson's *r*), indicating how they were calculated

Our web collection on statistics for biologists contains articles on many of the points above.

Software and code

Policy information about availability of computer code

Data collection	For CLIP-seq, the construction of small RNA libraries was performed using the SMARTer smRNA-Seq Kit for Illumina (Clontech). Sequencing was performed in 50 bp single-end mode using an Illumina HiSeq 2500 system (Illumina, CA, USA) and conducted by Macrogen Inc.<br>For mRNA-seq, the purified RNA samples were subjected to mRNA-seq library construction using the TruSeq Stranded mRNA Sample Preparation Kit (Illumina). The quality of the constructed cDNA libraries was validated according to the size distribution on an Agilent Bioanalyzer (DNA 1000 kit; Agilent) and quantitated by qPCR (Kapa Library Quant Kit; Kapa Biosystems, Wilmington, MA). The library was adjusted to 2 nmol/l for NGS on the Illumina HiSeq 2500 platform (100-base paired-end reads).<br>Light Cycler 480 ll machine was used for qRT-PCR. Confocal image was collected by Zeiss LSM 510 Meta and LSM800.
Data analysis	The raw data obtained from the high-throughput sequencing were preprocessed using Cutadapt (v1.12), riboPicker (v0.4.3).The filtered reads were mapped to the reference human genome (hg19) using the STAR software (v2.5.2b). The CLIP peaks were assigned to each exonic region using the intersectBed module from Bedtools (v2.25.0). The mapped reads from mRNA sequencing were quantified in FPKM using Cufflinks (v2.2.1). To estimate changes in the 3'UTR length, the QAPA (v1.0.0) was used. The peak calling of LC3B CLIP was performed using the piranha software (v.1.2.1). The motifs of CLIP peaks were analyzed using the HOMER package (v.4.8.3) and MEME (v.5.0.1). Using the htseq-count Python code (v0.6.11), the read counts per mRNA for half-life calculation were estimated. They were then normalized to count-per-million (CPM) value by using EdgeR (v3.12). The MFE was calculated by ViennaRna package (v2.4) Alignment of multiple amino acid sequences of LC3B among various species was performed by CLUSTALW from BioEdit v7.2.5 and visualized by ESPript3. The polarization for FP assay was calculated and drawn using GraphPad Prism 9.

For manuscripts utilizing custom algorithms or software that are central to the research but not yet described in published literature, software must be made available to editors and reviewers. We strongly encourage code deposition in a community repository (e.g. GitHub). See the Nature Research guidelines for submitting code & software for further information.

Data

Policy information about availability of data

All manuscripts must include a data availability statement. This statement should provide the following information, where applicable:

- Accession codes, unique identifiers, or web links for publicly available datasets
- A list of figures that have associated raw data
- A description of any restrictions on data availability

All raw sequencing data from this study have been submitted to the Sequence Read Archive under accession no. SRP303078.

Field-specific reporting

Please select the one below that is the best fit for your research. If you are not sure, read the appropriate sections before making your selection.

☒ Life sciences ☐ Behavioural & social sciences ☐ Ecological, evolutionary & environmental sciences

For a reference copy of the document with all sections, see nature.com/documents/nr-reporting-summary-flat.pdf

Life sciences study design

All studies must disclose on these points even when the disclosure is negative.

Sample size	All experiments were performed at least two times independently. More than 50 cells in each experiments were counted for quantitating confocal data.
Data exclusions	No data was excluded.
Replication	Completely independent experiments were carried out at least two independent replicates.
Randomization	No need for randomization of our samples because only cell-based experiments (no animals) were performed in this study.
Blinding	Samples were prepared by unblinded investigators but confocal analysis. Confocal analysis for quantitation was performed in blind at three times biological replicates.

Reporting for specific materials, systems and methods

We require information from authors about some types of materials, experimental systems and methods used in many studies. Here, indicate whether each material, system or method listed is relevant to your study. If you are not sure if a list item applies to your research, read the appropriate section before selecting a response.

Materials & experimental systems

n/a	Involved in the study	
☐	☒	Antibodies
☐	☒	Eukaryotic cell lines
☒	☐	Palaeontology and archaeology
☒	☐	Animals and other organisms
☒	☐	Human research participants
☒	☐	Clinical data
☒	☐	Dual use research of concern

Methods

n/a	Involved in the study	
☒	☐	ChIP-seq
☒	☐	Flow cytometry
☒	☐	MRI-based neuroimaging

Antibodies

Antibodies used	LC3B (Novus, NB100-2220; Cell Signaling Technology, #3868, 1:1000; or ORIGENE, AM20212PU-N, 1:50), CNOT1 (Proteintech, 14276-1-AP, 1:1000), CNOT7 (Abcam, ab195587, 1:1000), PRMT1 (Sigma-Aldrich, 07-404, 1:10000), FLAG (Sigma-Aldrich, A8592, 1:50; or Sigma-Aldrich, F3165, 1:1000), G3BP1 (Proteintech, 13057-2-AP, 1:10000), GFP (Santa Cruz Biotechnology, sc-9996, 1:50), U1 snRNP70 (Santa Cruz Biotechnology, sc-390899, 1:1000), β-actin (Sigma-Aldrich, A5441, 1:10000), and GAPDH (Ab Frontier, LF-PA0212, 1:10000). Peroxidase-conjugated goat anti-mouse IgG antibody (Sigma-Aldrich, AP124P), peroxidase-conjugated goat anti-rabbit IgG antibody (Sigma-Aldrich, AP132P), alexa Fluor ® 488–conjugated goat anti-mouse IgG antibody (Invitrogen, A11017) and rhodamine-conjugated goat anti-rabbit IgG antibody (Invitrogen, 31670).
Validation	All antibodies are all purchased from commercial manufacturers and all validated by the manufacturers. The validation can be checked in their website using the respective catalog number on the following website.<br>Novus (https://www.novusbio.com/)<br>Cell signaling (https://www.cellsignal.com/)

ORIGENE (https://www.origene.com/)
Proteintech (https://www.ptglab.com/)
Abcam (https://www.abcam.com/)
Sigma-Aldrich (https://www.sigma.com/)
Santa Cruz Biotechnology (https://www.scbt.com)
Ab Frontier (http://www.younginfrontier.com/laboratory/abfrontier/)
Invitrogen (https://www.thermofisher.com/)

Eukaryotic cell lines

Policy information about cell lines

Cell line source(s)	HEK293T and HeLa cells are purchased from ATCC. HeLa cells stably expressing GFP-LC3B are provided by Prof. Chungho Kim (Korea university, Seoul, Korea).
Authentication	HEK293T and HeLa cells were purchased and authenticated from ATCC, bioresource center.
Mycoplasma contamination	MycoAlert PLUS Mycoplasma detection kit was used for ensure that the cells have no mycoplasma contamination.
Commonly misidentified lines (See ICLAC register)	No commonly misidentified lines in this study.
